# Supplementary material for: Antioxidants Isolated from Elaeagnus umbellata (Thunb.) Protect against Bacterial Infections and Diabetes in Streptozotocin-Induced Diabetic Rat Model
Source: Molecules. 2021 Jul 24;26(15):4464. doi: 10.3390/molecules26154464 (PMC8348310; doi:10.3390/molecules26154464)
Supplement: Supplementary file 1 [file molecules-26-04464-s001.zip › molecules-1273891-supplementary.pdf]

molecules

Supplementary file

Antioxidants Isolated from *Elaeagnus umbellata* (Thunb.) Protect Against Bacterial Infections and Diabetes in Streptozotocin-Induced Diabetic Rat Model

Nausheen Nazir [1], Muhammad Zahoor [1,*], Mohammad Nisar [2], Imran Khan [3], Riaz Ullah [4] and Amal Alotaibi [5,*]

1 Department of Biochemistry, University of Malakand, Chakdara 18800, Khyber Pakhtunkhwa, Pakistan; nausheen.nazir@uom.edu.pk
2 Department of Botany, University of Malakand, Chakdara 18800, Khyber Pakhtunkhwa, Pakistan; mnshaalpk@yahoo.com
3 Department of Pharmacy, University of Swabi, Anbar 94604, Khyber Pakhtunkhwa, Pakistan; drimran.khan@hotmail.com
4 Department of Pharmacognosy, (MAPPRC), College of Pharmacy, King Saud University, Riyadh 11451, Saudi Arabia; rullah@ksu.edu.sa
5 Basic Science Department, College of Medicine, Princess Nourah bint Abdulrahman University, Riyadh 11671, Saudi Arabia
* Correspondence: authors: mohammadzahoorus@yahoo.com (M.Z.); amaalotaibi@pnu.edu.sa (A.A.)

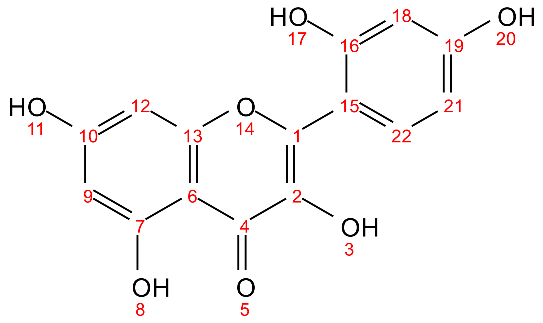

Figure S1. Structural formula of compound-I (Morin).

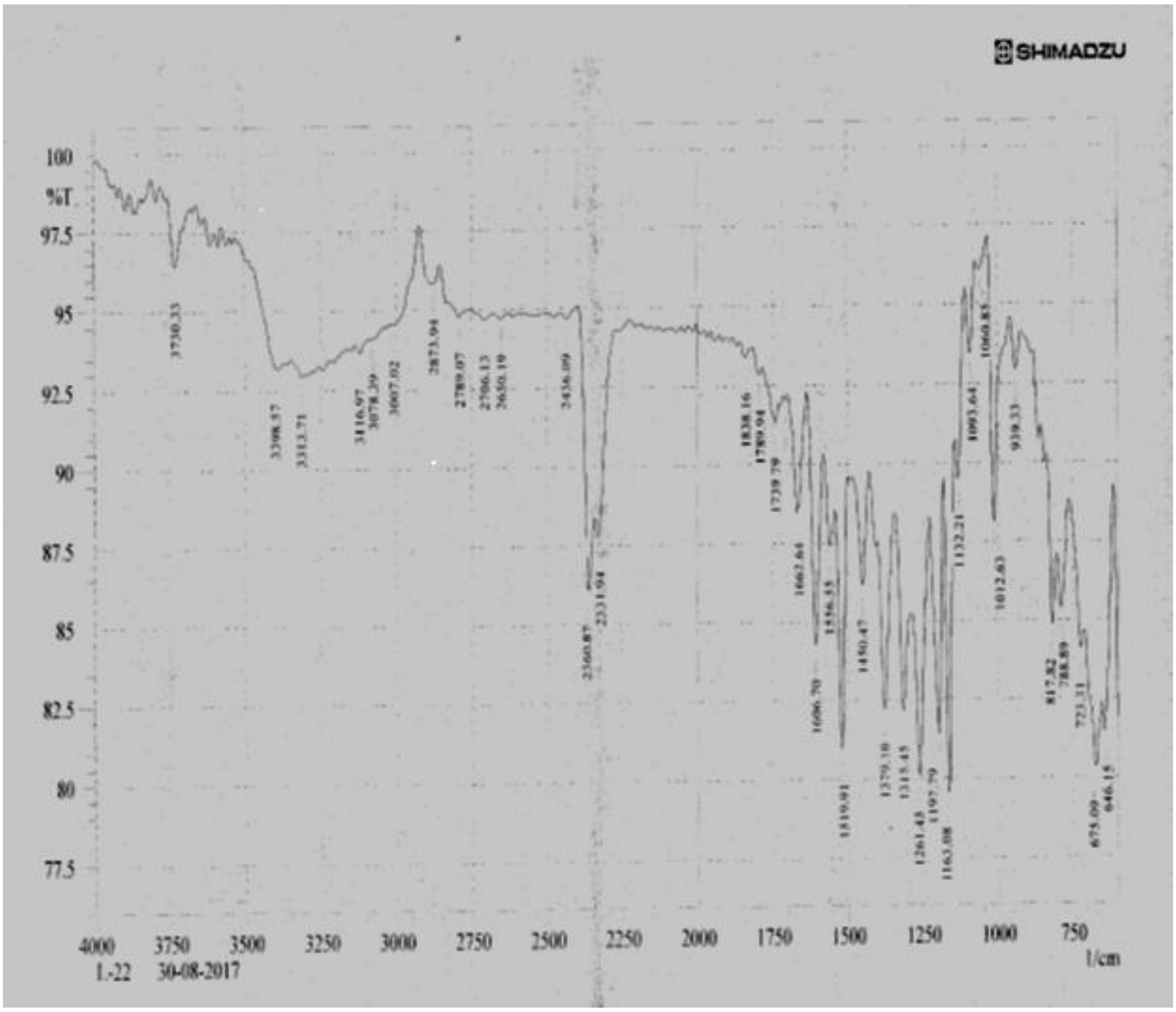

Figure S2. FTIR spectra of compound-**I** (Morin).

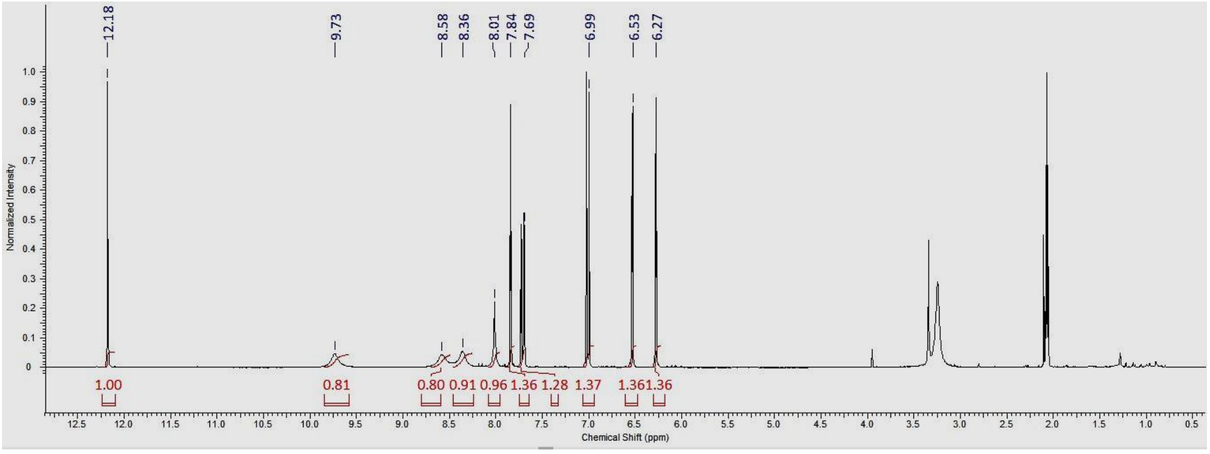

Figure S3. ^{1}H NMR (Acetone, 300MHz) of compound-**I** (Morin).

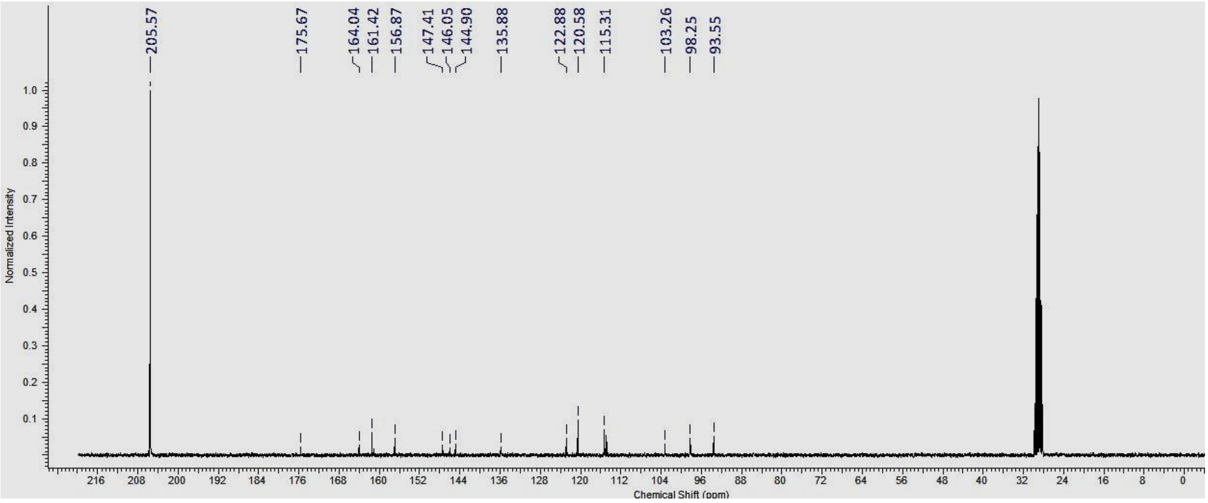

Figure S4. ^{13}C NMR (Acetone, 75MHz) of compound-I (Morin).

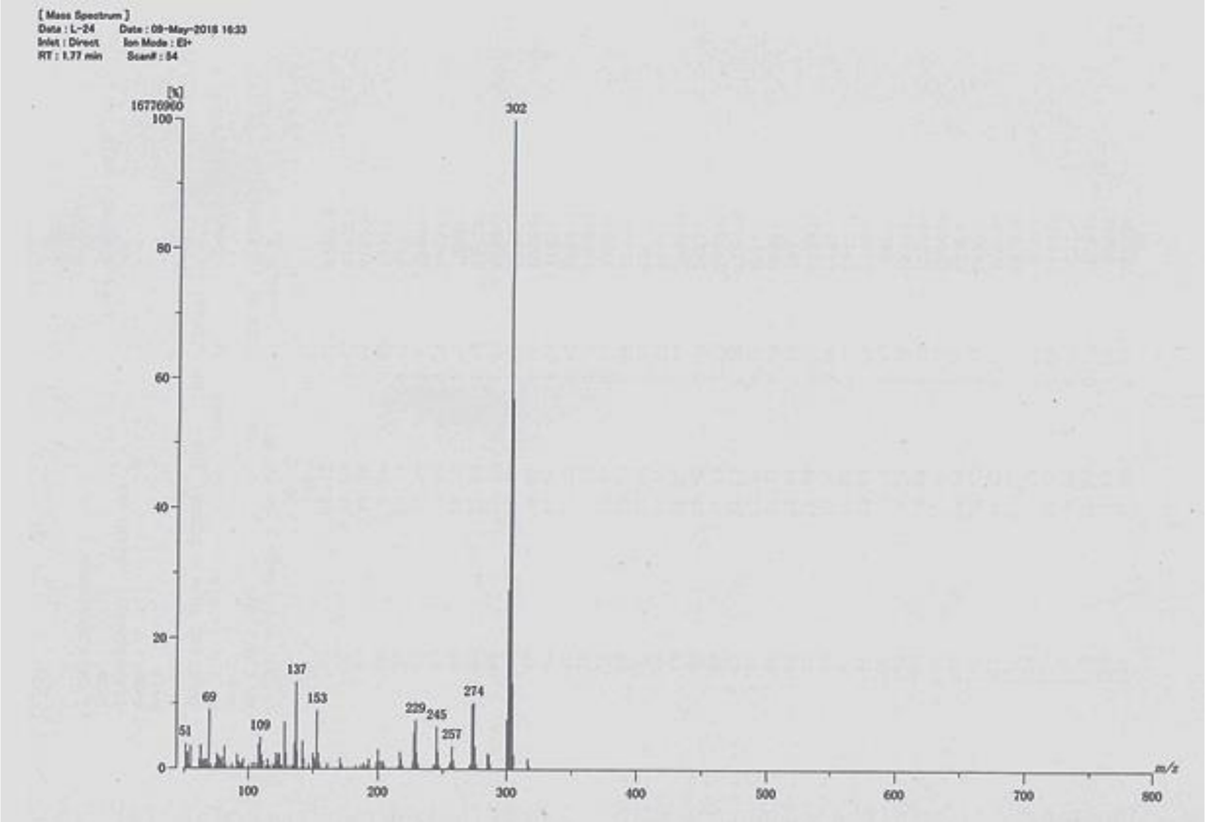

Figure S5. EIMS of compound-I (Morin).

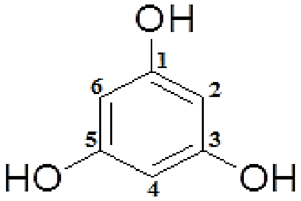

Figure S6. Structural formula of compound-**II** (Phloroglucinol).

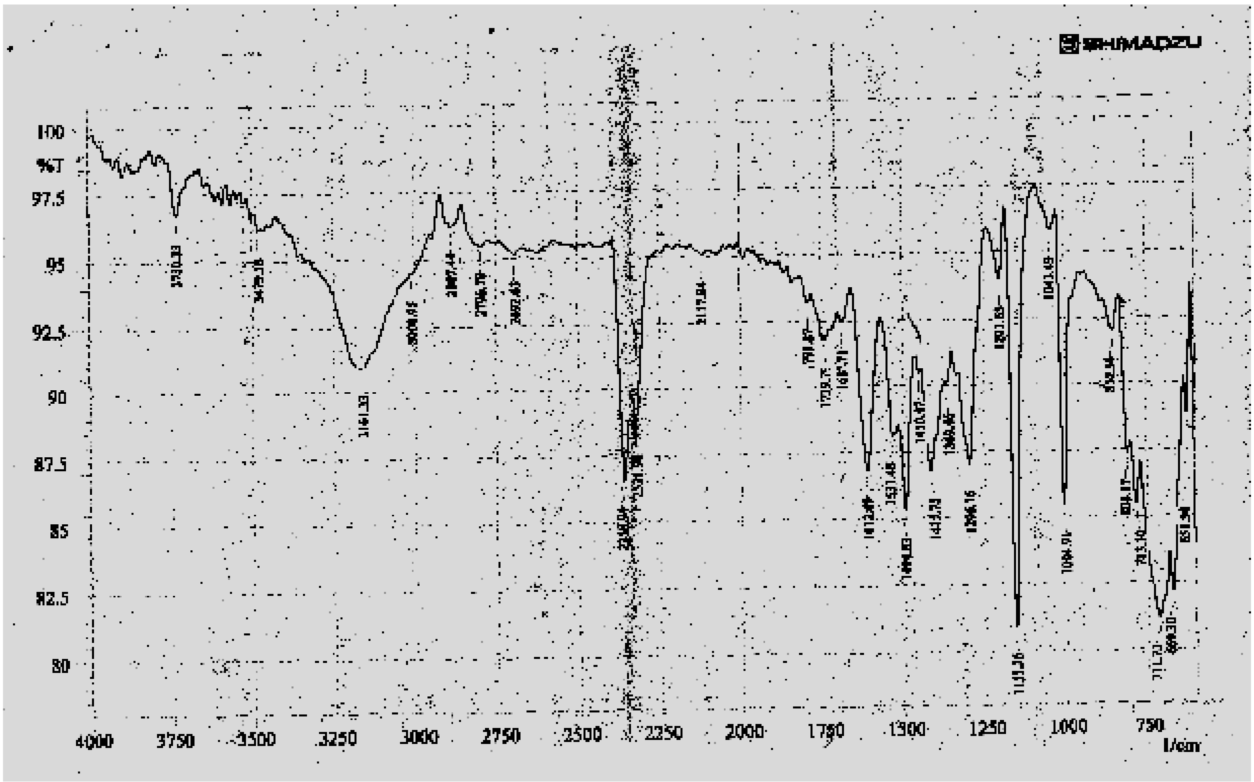

Figure S7. FTIR spectra of compound-**II** (Phloroglucinol).

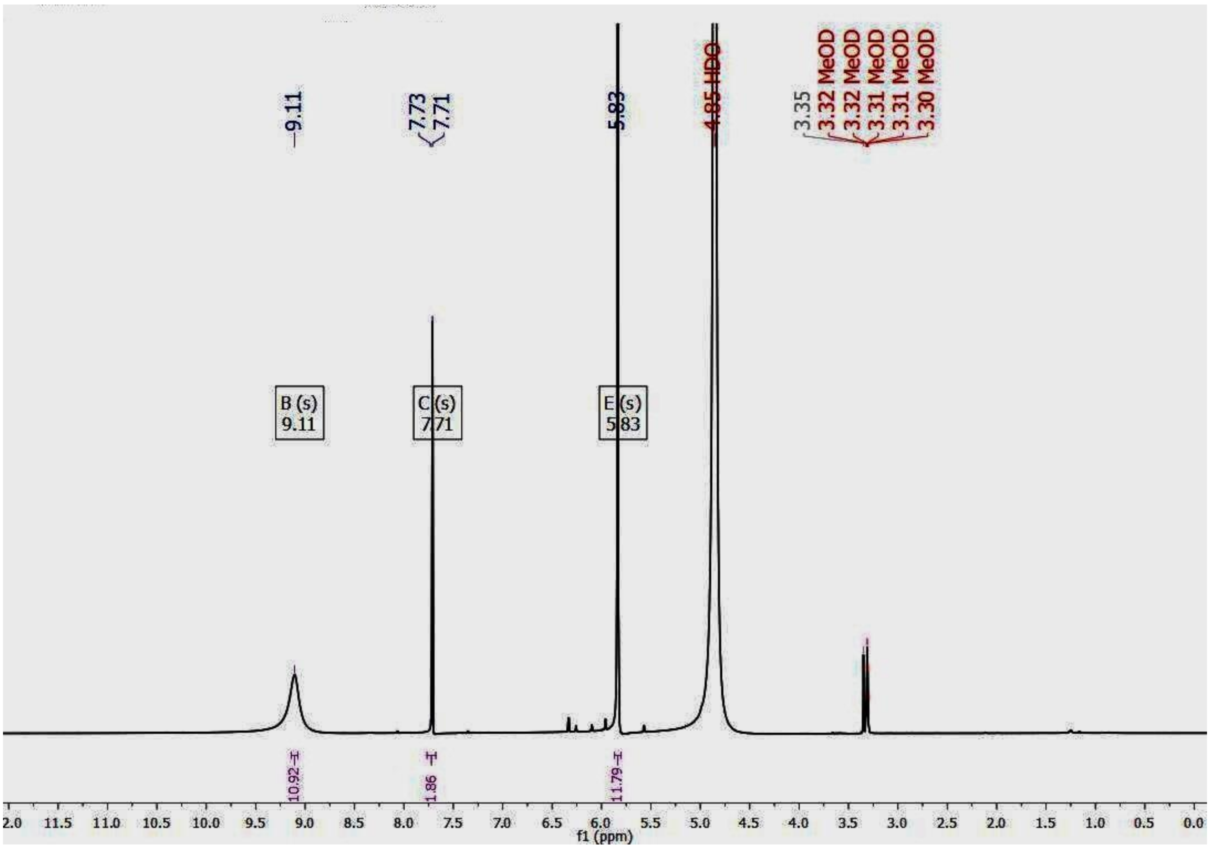

Figure S8. 1H- (Methanol, 500 MHz) of compound-**II** (Phloroglucinol).

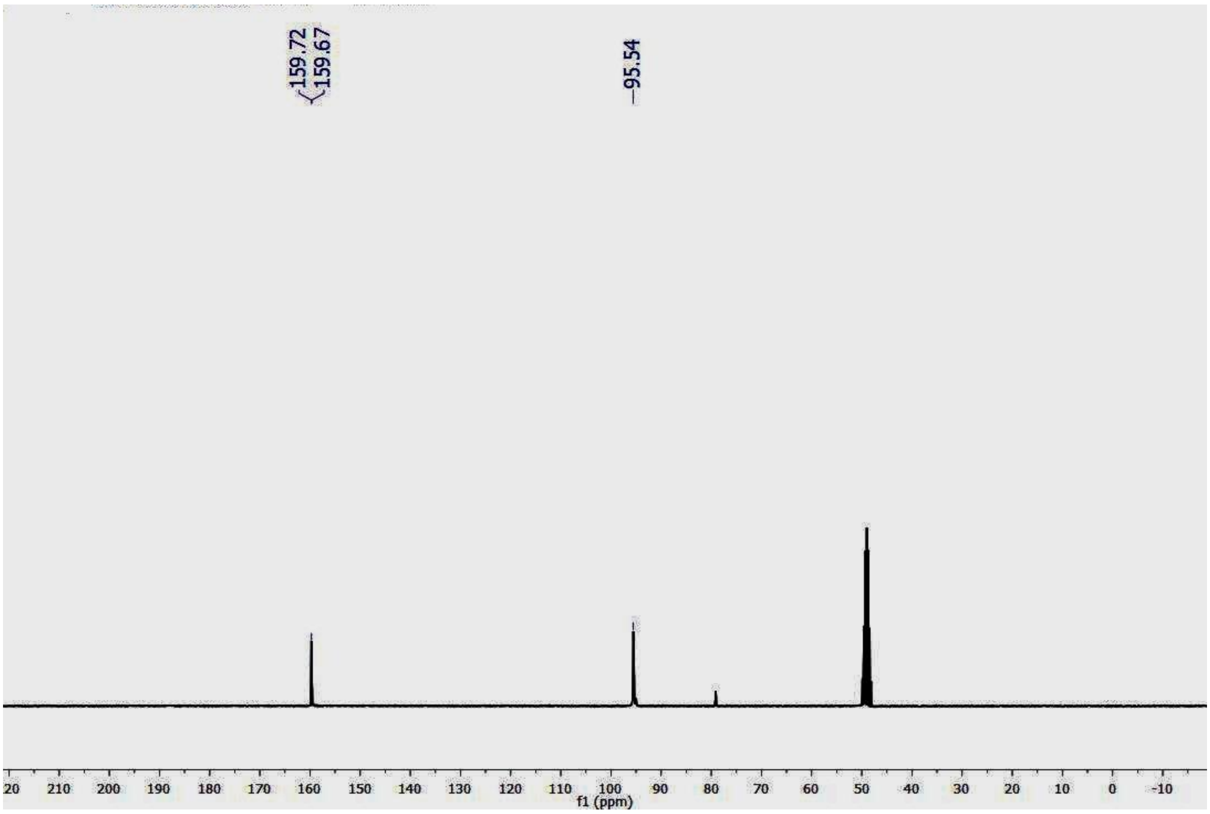

Figure S9. ^{13}C-NMR (Methanol, 126 MHz) of compound-**II** (Phloroglucinol).

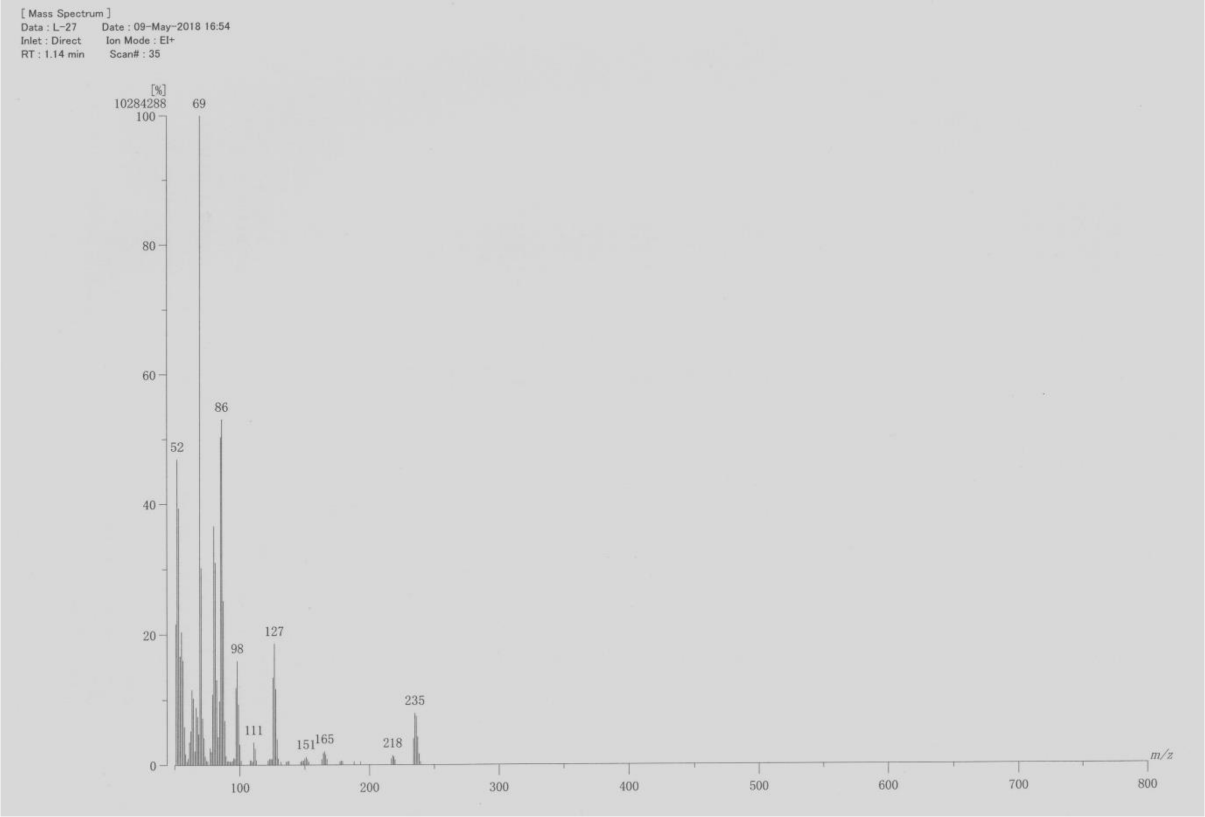

Figure S10. EI-MS Mass of compound-**II** (Phloroglucinol).

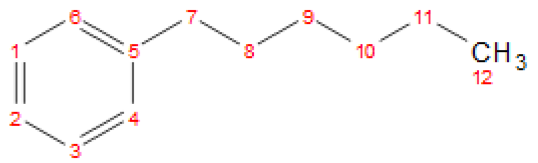

Figure S11. Structural formula of compound-**III** (1-Hexyl benzene).

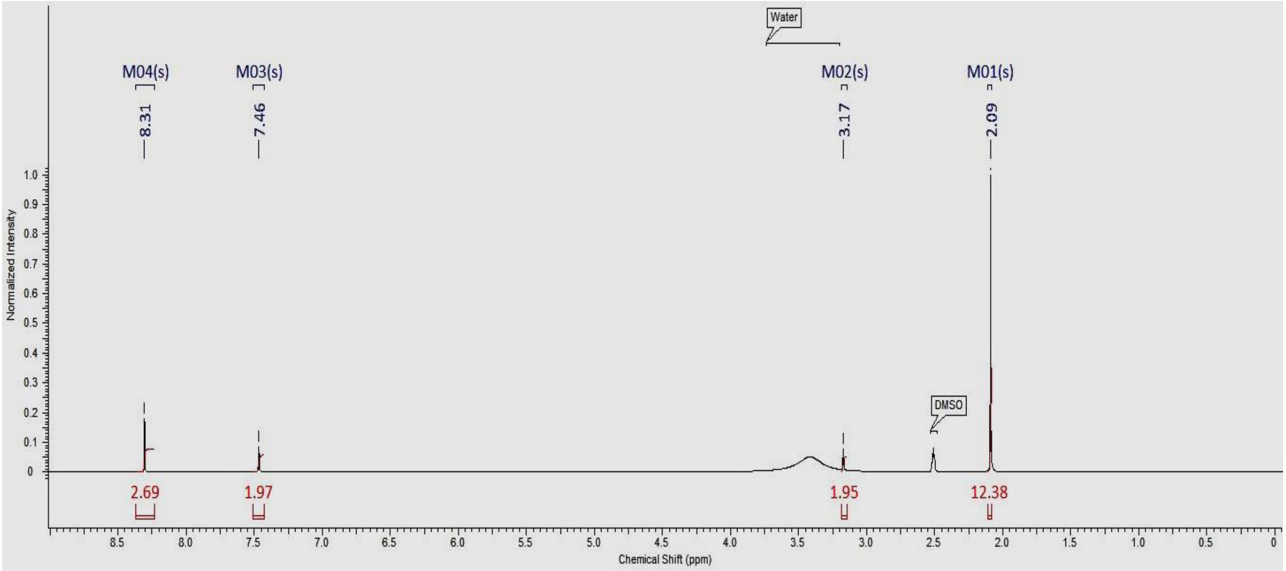

Figure S12. ^{1}H NMR (DMSO- H_2O, 300MHz) of compound-**III** (1-Hexyl benzene).

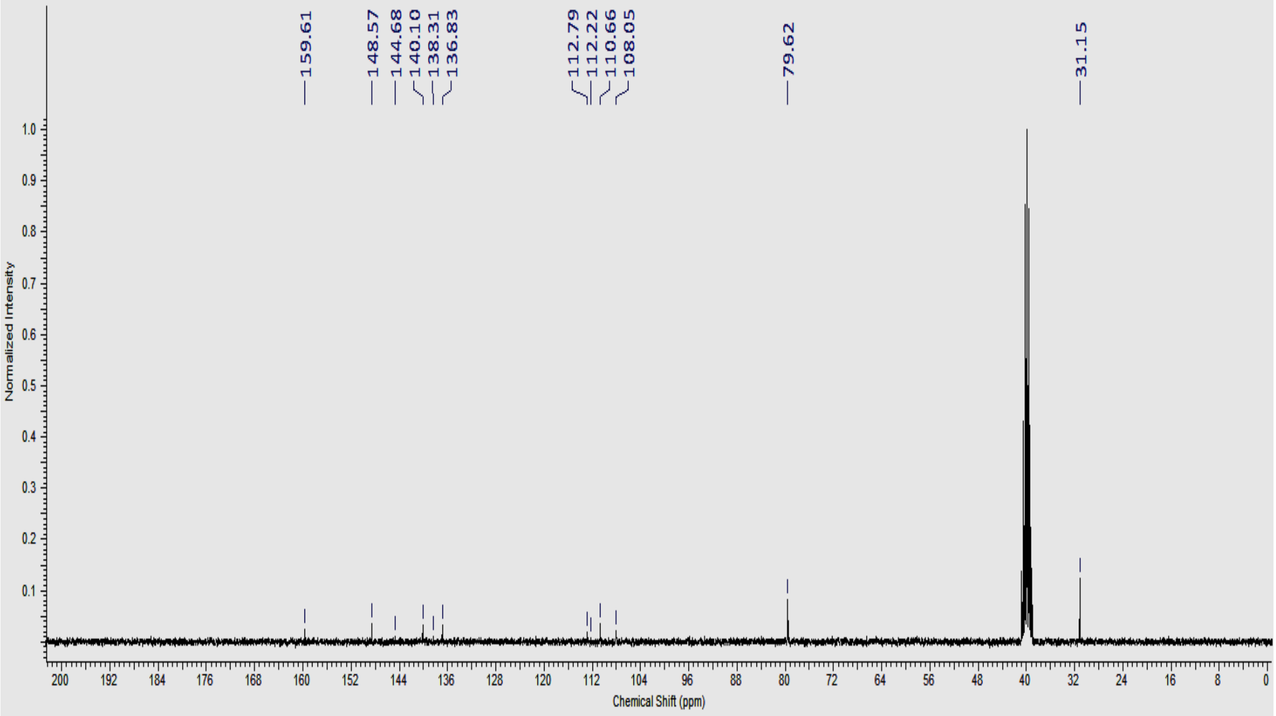

Figure S13. ^{13}C NMR (DMSO-d6, 75MHz) of compound-**III** (1-Hexyl benzene).

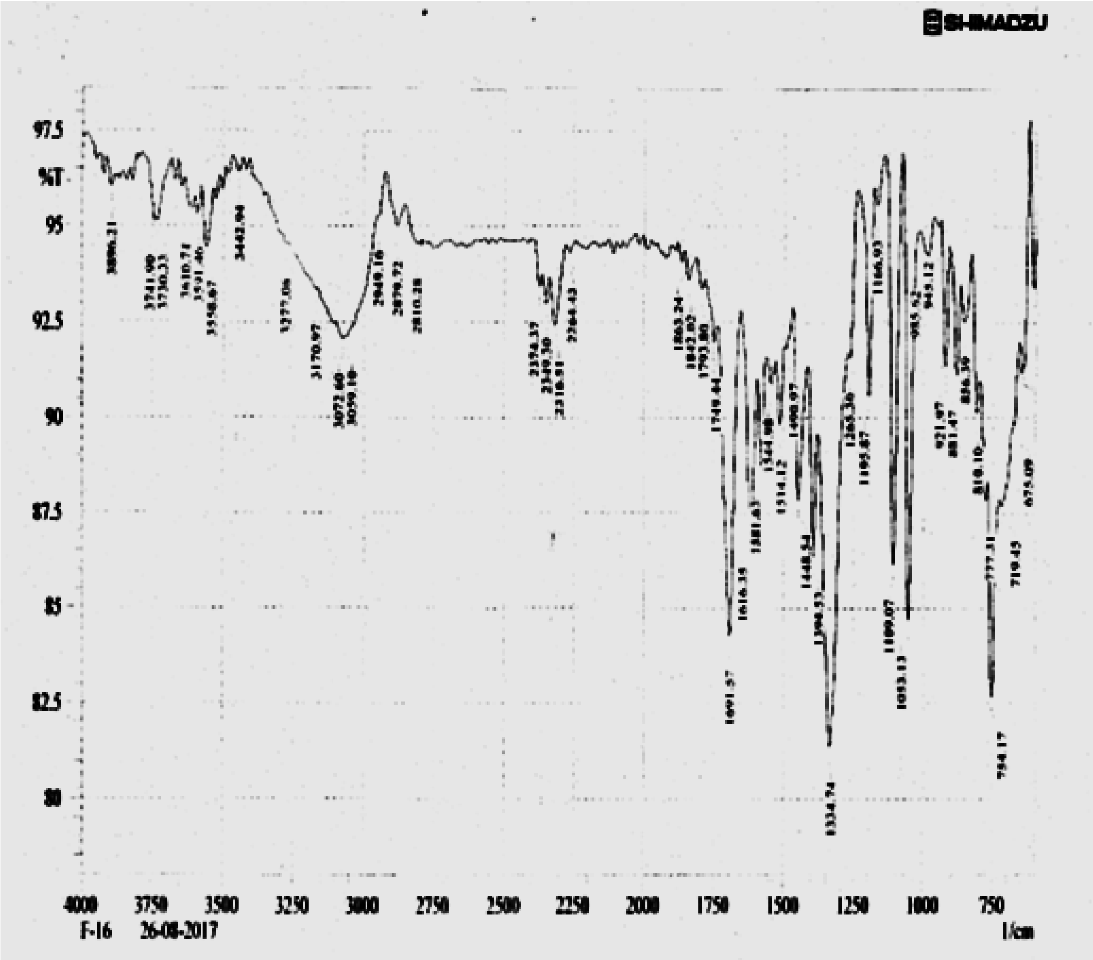

Figure S14. FTIR spectra of compound-**III** (1-Hexyl benzene).

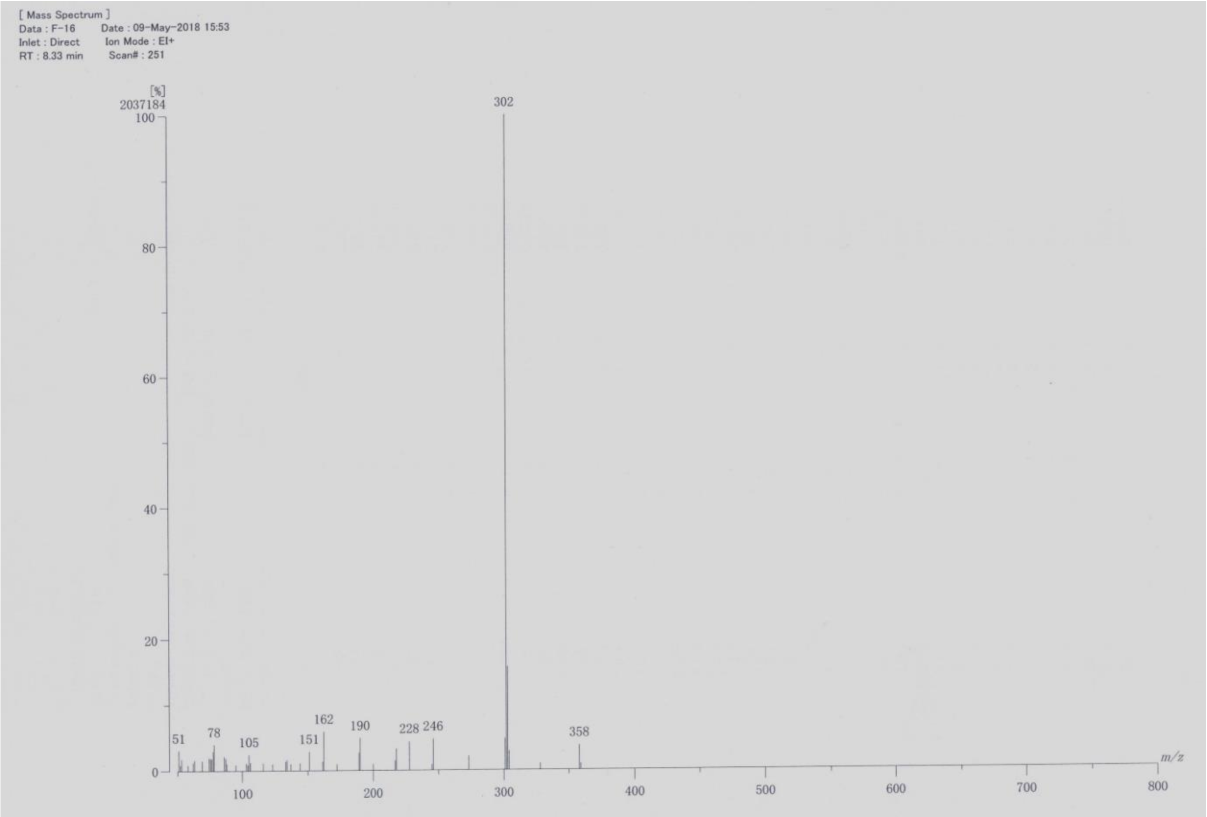

Figure S15. EIMS of compound-**III** (1-Hexyl benzene).
